# Early injury of the neonatal lung contributes to premature lung aging: a hypothesis

**DOI:** 10.1186/s40348-016-0052-8

**Published:** 2016-07-12

**Authors:** Silke Meiners, Anne Hilgendorff

**Affiliations:** Comprehensive Pneumology Center (CPC), Ludwig-Maximilians University, Helmholtz Zentrum München, German Center for Lung Research (DZL), Max-Lebsche-Platz 31, 81377 München, Germany; Perinatal Center Grosshadern, Dr. von Haunersches Children’s Hospital, Ludwig-Maximilians University, Munich, Germany

**Keywords:** BPD premature aging, Hyperoxia, Early injury, Immature lung

## Abstract

Chronic lung disease of the newborn, also known as bronchopulmonary dysplasia (BPD), is the most common chronic lung disease in early infancy and results in an increased risk for long-lasting pulmonary impairment in the adult. BPD develops upon injury of the immature lung by oxygen toxicity, mechanical ventilation, and infections which trigger sustained inflammatory immune responses and extensive remodeling of the extracellular matrix together with dysregulated growth factor signaling. Histopathologically, BPD is characterized by impaired alveolarization, disrupted vascular development, and saccular wall fibrosis. Here, we explore the hypothesis that development of BPD involves disturbance of conserved pathways of molecular aging that may contribute to premature aging of the lung and an increased susceptibility to chronic lung diseases in adulthood.

## Introduction

Chronic lung disease of the newborn, also known as bronchopulmonary dysplasia (BPD), is the most common chronic lung disease in early infancy and results in an increased risk for pulmonary and neurologic impairment persisting into adulthood [1]. BPD is defined by the need for supplemental oxygen and/or ventilator support for longer than 28 days, or beyond 36 weeks post-menstrual age, and is classified into three different grades of severity (mild, moderate, severe) [2]. The incidence of BPD is reported up to 77 % in infants born at less than 32 weeks of gestation with a birth weight below 1 kg [3]. Histopathologically, BPD lungs show impaired alveolarization associated with diminished development of small vessels [4, 5]. These structural alterations are accompanied by characteristic inflammatory changes and extensive remodeling of the extracellular matrix (ECM) together with increased smooth muscle mass in small pulmonary arteries and airways [4]. Risk factors for the development of BPD that have been identified by clinical and experimental studies include infections occurring both in utero and post-partum, as well as oxygen toxicity and the impact of mechanical ventilation [6]. Such injuries occur beyond structural and functional immaturity of the organ and the background of genetic susceptibility.

Below, we would like to explore the hypothesis that early lung injury affects conserved pathways of aging thereby contributing to the development of BPD. For that, we first outline the molecular pathways of aging and then summarize available knowledge on how these pathways are affected by experimental hyperoxia and mechanical ventilation of the newborn lung and in BPD. Recent data indicate that adult preterm birth survivors, especially those who developed BPD, exhibit features of clinically relevant respiratory dysfunction later in life [7, 8]. We propose that early alterations in major aging pathways drive premature aging of the lung thereby adding to the risk for development of chronic lung diseases later in life [9, 10].

## Review

### Molecular concepts of aging

Over the past 30 years, basic and translational research has identified several molecular pathways of aging defined as the “hallmarks of aging,” i.e., genomic instability, telomere attrition, epigenetic alterations, loss of proteostasis, deregulated nutrient sensing, mitochondrial dysfunction, cellular senescence, altered intercellular communication, and stem cell exhaustion, which provide a molecular foundation for organismal aging [11]. Strikingly, all of these pathways are key pathways for organismal growth, maintenance, and communication. We have recently added an additional hallmark to these molecular pathways, i.e., dysregulation of the ECM, and dissected the distinct aging hallmarks for their differential contribution to the development of age-related chronic lung diseases such as chronic obstructive pulmonary disease (COPD), lung cancer, and idiopathic pulmonary fibrosis (IPF) [12]. Below, we will summarize available evidence that early injury of the neonatal lung as in BPD affects distinct hallmark pathways of aging. This may drive premature aging of the adult lung and early onset of chronic lung diseases in later life.

### Molecular pathways of aging are altered by injury of the neonatal lung and in BPD

Hyperoxia as mediated by oxygen supplementation results in increased levels of reactive oxygen species and subsequent oxidative damage of DNA contributing to genomic instability [13, 14]. Very similar to the adult lung, oxygen supplementation of preterm infants induces oxidative stress in the immature lung and causes oxidative modifications of DNA and activation of DNA damage response pathways such as p53 and ATM as observed in hyperoxic ventilated premature baboons and in a rat model of BPD [15–18]. In spontaneous dwarf rats, increased resistance to hyperoxic stress was associated with reduced signs of DNA damage in multiple organs including the lung and contributed to an extended life span of this rat strain compared to their wild-type controls [19]. These data suggest a causal relationship between regulation of DNA damage upon hyperoxia and life span.

Telomere sequences protect the ends of chromosomes and are lost upon DNA replication, thereby limiting the proliferative capacity of cells [20]. Attrition of telomeres over time is a characteristic feature of aging and serves as one of the major markers for premature aging [11]. While there is no evidence for a general association of birth size and telomere length in adult life [21], some recent studies suggest that telomere length in circulating leukocytes or salivary cells is shorter in young adults born preterm compared to that in young adults born at term [22, 23]. Accelerated attrition of telomeres may thus potentially add to the risk of chronic lung diseases as development and progression of both, COPD and IPF, have been associated with reduced telomere function [12].

Development and maturation of the neonatal to the adult lung involves major epigenetic alterations such as changes in DNA methylation, histone marks, and microRNA (miRNA) expression [24–29]. Emerging evidence suggests that early alterations in epigenetic marks are associated with chronic lung diseases such as asthma, COPD, and IPF [30]. Dysregulation of chromatin remodeling pathways including DNA methylation, histone acetylation, and miRNA regulation have also been reported in response to hyperoxia in the neonatal lung in several experimental models and in BPD patients: The process of alveolar septation in the mouse and human lung is accompanied by altered DNA methylation profiles that coincide with distinct changes in gene expression. Of note, dysregulated alveolar septation as observed in BPD was associated with distinct methylation profiles suggesting that abnormal DNA methylation contributes to differential gene expression in human BPD [31]. In particular, bone morphogenetic protein (BMP) 7 showed an inverse correlation between DNA methylation and expression in human BPD samples, with BMP7 being expressed at reduced levels in BPD. As BMP7 opposes the activity of transforming growth factor beta (TGF-β), this may allow enhanced TGF-β signaling in the immature lung contributing pulmonary fibrosis and arrested lung development [32]. Alterations in chromatin remodeling and histone acetylation have also been reported for preterm infants that were at risk for BPD development [33]. In experimental rat and mouse models of neonatal lung injury, hyperoxia was shown to result in diminished expression of histone deacetylases (HDAC) 1 and 2 similar to reduced HDAC activity in lungs of COPD patients but contrary to the increased levels of HDACs in IPF lungs [34–37]. In hyperoxia-treated neonatal rats, DNA methylation by DNMT3b- and EZH2-catalyzed histone methylation was observed [38]. Whether similar DNA and histone modifications also occur in chronic lung diseases remain to be investigated. Pronounced changes in the miRNA profile have also been observed in response to hyperoxia in neonatal lungs during experimental development of BPD [39, 40]. A recent meta-analysis on miRNA profiles in BPD reported upregulation of miRNA-21, miRNA-34a, miRNA-431, and Let-7f and downregulation of miRNA-335 in BPD lung tissues compared to normal groups [41]. Except for miRNA-21, however, there is no major overlap with miRNA profiles of patients with chronic lung diseases [42], suggesting that miRNA regulation in the newborn lung might be inherently different from the adult organ [43]. In summary, these experimental and clinical data suggest that changes in epigenetic programming are associated with neonatal lung damage by oxygen supplementation and BPD development but that there are inherent differences between the immature newborn and adult lung.

Proteostasis, i.e., maintenance of protein homeostasis, ranges from correct protein synthesis, proper protein maturation, folding, and interaction to controlled disposal of unwanted and damaged proteins. Loss of proteostasis has been proposed as a major hallmark of aging [11]. Accumulating data suggest that protein folding and degradation pathways are dysregulated by hyperoxia and mechanical ventilation as recently reviewed by us [44]. For the newborn lung, some evidence suggests that increased stress of the endoplasmic reticulum (ER) [45] and augmented autophagy contributes to hyperoxia-induced surfactant protein (SP)-C accumulation and subsequent injury of neonatal rat lungs [46]. Increased ER stress and autophagy have also been described as characteristic features of IPF and COPD, respectively [47]. Thorough evaluation of protein homeostasis in the preterm lung, however, is missing possibly due to the difficulties in obtaining reliable protein data from small-sized lung samples in experimental and clinical BPD.

Another aging hallmark is deregulated nutrient sensing via the IGF-1/AKT/mTOR axis, an evolutionary conserved growth signaling pathway that integrates nutrient signals to regulate cell growth [48]. For BPD, intrauterine growth restriction that results from nutritional and hormonal (e.g., insulin) deficiencies of the fetus represents an independent risk factor [49, 50]. Insufficient nutrient supply associated with reduced levels of insulin impairs pulmonary alveolar and vessel growth in fetal sheep lungs [51]. On the cellular level, there seems to be a fine-tuned balance of mTOR/Akt activation that needs to be maintained for proper lung maturation: On the one hand, induction of Akt signaling protected neonatal lungs from hypoxia-induced injury [52], while inactivation of the mTOR/Akt pathway negatively regulated SP-A secretion in alveolar epithelial cells and contributed to respiratory distress syndrome in mice or early lethality, respectively [53, 54]. Thus, while the worsening effects of diminished nutrient supply are well established for BPD development, there is only little known about how growth factor signals are integrated via the IGF-1/AKT/mTOR signaling axis and how signaling is regulated in a cell type-specific manner in BPD.

Exhaustion of stem cells has been implicated as a driving factor for several age-related diseases [55, 56]. Most probably, exhaustion of stem cells is not a typical feature of BPD, but impaired mobilization of bone marrow-derived progenitor cells and/or increased sensitivity to oxidative stress might be a contributing factor to BPD disease pathology [57, 58]. Accordingly, reduced recruitment of endothelial progenitor cells (EPC) from the bone marrow has been reported in neonatal mice in response to hyperoxia while adult mice showed rather increased EPC levels [59]. Moreover, reduced numbers of cord vein-derived progenitor cells have been associated with development of BPD [60, 61]. The concept of protective effects of progenitor and stem cells is supported by two recent studies that showed that paracrine effects of exogenously supplemented stem cells alleviate impaired alveolar growth in neonatal lung injury in rats and mice [62, 63]. Therapeutic application of stem cells, e.g., mesenchymal stem cells (MSC), for treatment of BPD is thus a promising option [58, 64, 65].

Mitochondrial dysfunction has been proposed as another hallmark of aging [11]. The free radical theory of aging early on proposed that dysfunctional mitochondria contribute to elevated levels of reactive oxygen species (ROS) and subsequent oxidative damage of the cell [66]. This concept has been extended in recent years to a more complex understanding of mitochondrial metabolism and mitochondrial DNA function in aging [67]. Hyperoxia has been shown to impair both glycolysis and oxidative phosphorylation in alveolar epithelial cells contributing to elevated ROS formation [68]. In the newborn lung, hyperoxia treatment impaired mitochondrial respiration and added to BPD development. Moreover, direct inhibition of oxidative phosphorylation by pyraben, an inhibitor of mitochondrial complex I, resulted in abnormal alveolar development [69]. Another study implicated that only early hyperoxia-induced postnatal mitochondrial ROS production contributes to BPD development [70]. Mitochondrial dysfunction, as, e.g., induced by hyperoxia, may thus contribute to early lung damage of the neonatal lung and development of BPD.

Cellular senescence is a defined cellular program of aging that limits the replicative capacity of cells by cell cycle arrest, thereby preventing propagation of old and damaged cells [71]. Senescence also contributes to tissue remodeling during embryonic development and upon tissue damage [72]. In the neonatal mouse lung, the senescence program was activated by hyperoxia via decreased histone deacetylase activity and upregulation of p21, contributing to impaired alveolarization [34]. The impact of prenatal cigarette smoke on premature senescence may be explained by caveolin-1 expression, linking lung fibroblast senescence and development of pulmonary emphysema [73].

Altered intercellular communication due to disturbed cell-cell signaling is another characteristic feature of aging [11]. The release of cytokines and imbalanced growth factor signaling in the preterm lung, e.g., TGF-β, leads to the activation of different transcription factors and results in a characteristic increase in apoptosis of different cell types [74]. In particular, platelet-derived growth factor-α and the fibroblast growth factor family play a major role in myofibroblast-driven secondary crest formation [75]. The presence of dysmorphic capillaries is related to an altered pattern of angiogenic growth factors such as reduced expression of the vascular endothelial growth factor and its receptors in the neonatal lung [76, 77], accompanied by diminished endothelial nitric-oxide synthase and soluble guanylate cyclase in lung blood vessels and airways [78, 79]. These changes contribute to subsequent development of pulmonary hypertension and impaired lung lymphatic drainage [80]. Perturbation of central signaling pathways, e.g., suppression of the nuclear factor kappa B (NF-kB), disrupts airway-branching and impairs development of epithelial, mesenchymal, and endothelial cell structures culminating in a failure of lung development [81–83]. Moreover, there is altered communication of immune and inflammatory cells with parenchymal cells of the immature lung contributing to innate and adaptive immune responses in BPD [84, 85]. These data clearly indicate that altered intercellular communication as one of the aging hallmarks is of central relevance for the development of BPD and most likely contributes to long-term consequences as the lung matures and ages.

We have recently proposed dysregulation of the ECM as another pillar in the process of lung aging [12]. Similar to disease-relevant processes in the aging adult lung, ECM dysregulation is a characteristic feature of the injured neonatal lung [86]. Experimental studies in rodents and baboons recapitulated ECM alterations and clearly linked ECM remodeling to apoptosis, inflammation, and altered growth factor signaling [85, 87–91]. Accordingly, therapeutic prevention of ECM degradation in the neonatal mouse lung preserved lung growth and structure upon mechanical ventilation [92, 93]. Moreover, the ECM is more than a simple scaffold but also directs the fate of cellular differentiation. This was convincingly shown by several studies which re-populated cells in de-cellularized lungs of different matrix compositions [94–96]. Sustained reorganization of the ECM as observed in BPD may thus not only alter the function of the ECM as a scaffold for lung development but may also affect its potential to regulate cellular differentiation in the lung. As such, dysregulation of the ECM in BPD may contribute to an altered long-term ECM memory that fosters development of chronic lung diseases such as COPD and IPF [12].

## Conclusions

We are only beginning to understand the molecular pathways that contribute to the development of BPD. Despite some serious lack of knowledge, there is a remarkable overlap between cellular pathways involved in aging and BPD development, such as genomic instability, epigenetic alterations, loss of proteostasis, deregulated nutrient sensing, mitochondrial dysfunction, cellular senescence, altered intercellular communication, and ECM remodeling. Regarding the contribution of stem cells, some evidence suggests that instead of stem cell exhaustion, impaired mobilization and recruitment of progenitor cells may contribute to the development of BPD. The contribution of telomere attrition still remains to be investigated. Of note, most of the studies summarized here provide mainly descriptive evidence for an involvement of specific aging pathways in BPD. In order to identify a causal role of distinct aging pathways in the pathogenesis of BPD, one should test the effect of hyperoxia and mechanical ventilation in animal models with genetic modifications of single-pathway components: If these pathways causally contribute to the development of BPD, experimental aggravation would then accelerate damage of the neonatal lung while its experimental amelioration would retard BPD development. The hypothesis that early injury via specific impairment of aging pathways promotes age-related lung damage later in life can also be tested in such experimental models by analyzing lung function over time in adult animals after early injury and upon second hits such as cigarette smoke exposure or infections.

We recently proposed that the essential nature of the hallmarks of aging for the organism makes it very likely that one or the other pathway will be dysregulated in any chronic lung disease [12]. This notion may also apply to the development of BPD. Early dysregulation of cellular and tissue-related maintenance mechanisms such as DNA repair, proteostasis, stem cells, and cell/cell and cell/matrix interactions will then contribute to amplification of cellular damage over time and contribute to impaired lung function later in life. Therapeutic targeting of such central maintenance hubs may thus represent a promising mechanism to interfere with early injury-induced development of chronic lung diseases.

## Abbreviations

BPD, bronchopulmonary dysplasia; COPD, chronic obstructive pulmonary disease; ECM, extracellular matrix; IPF, idiopathic pulmonary fibrosis; NF-kB, nuclear factor kappa B; ROS, reactive oxygen species; SP, surfactant protein; TGF-β, transforming growth factor beta
